# The Impact of Artificial Intelligence-Supported Instruction on Student Learning in STEM: A Systematic Review and Meta-Analysis

**DOI:** 10.3390/jintelligence14060109

**Published:** 2026-06-15

**Authors:** Yunus Doğan, Zeynep Kılıç, Yusuf Kalınkara, Tarık Talan

**Affiliations:** 1 Foreign Languages and Tourism Faculty, Dicle University, Diyarbakir 21280, Türkiye; 2 Department of Radio, Television and Cinema, Mardin Artuklu University, Mardin 47200, Türkiye; zeynepkilic@artuklu.edu.tr; 3 Vocational School of Technical Sciences, Gaziantep Islam Science & Technology University, Gaziantep 27010, Türkiye; yusufkalinkara@gmail.com; 4 Faculty of Engineering and Natural Sciences, Gaziantep Islam Science & Technology University, Gaziantep 27010, Türkiye; ttalan46@hotmail.com

**Keywords:** artificial intelligence, STEM education, meta-analysis, student achievement, adaptive learning, intelligent tutoring systems

## Abstract

The rapid integration of artificial intelligence (AI) technologies into educational contexts has introduced innovative instructional approaches, particularly within Science, Technology, Engineering, and Mathematics (STEM) education. Although an increasing number of empirical studies have examined AI-supported instruction, existing findings remain heterogeneous, making it difficult to draw firm conclusions about its overall effectiveness. This study aims to systematically synthesize experimental and quasi-experimental research on AI-supported instructional interventions in STEM education, quantify their overall effects on student learning outcomes, and examine potential moderating factors, including educational level, STEM discipline, and intervention duration. A comprehensive systematic literature search was conducted across Web of Science, Scopus, ERIC, ScienceDirect, and Google Scholar, covering studies published between 2005 and 2025. A total of 35 studies meeting predefined inclusion criteria were included in the meta-analysis. Effect sizes were calculated using Hedges’ g, and a Random Effects Model (REM) was employed to account for heterogeneity among studies. Moderator analyses were conducted for educational level, STEM discipline, and intervention duration. Publication bias was assessed using multiple diagnostic methods. The meta-analysis revealed a statistically significant overall positive effect of AI-supported instruction on student learning outcomes in STEM education (g = 0.67, 95% CI [0.49, 0.85], *p* < 0.001). Moderator analyses indicated that AI interventions were most effective at the high school level. Although Science and Mathematics disciplines showed slightly higher effect sizes, the between-group difference was not statistically significant (Q = 4.85, df = 2, *p* = 0.088). Regarding intervention duration, the highest effect size was observed in interventions lasting more than one month and up to two months, though no consistent pattern of increasing effectiveness with longer durations was found. Publication bias analyses suggested minimal influence on the overall findings. AI-supported instructional interventions demonstrate a moderately to highly positive impact on student learning outcomes in STEM education. The effectiveness of these interventions varies according to educational level, disciplinary context, and intervention duration. These findings provide robust empirical evidence supporting the pedagogical value of AI in STEM education and offer guidance for educators and policymakers regarding effective implementation.

## 1. Introduction

The rapid technological transformation of the 21st century is profoundly reshaping multiple domains, with education being one of the most affected sectors. Traditional teaching methods, which often rely on static lectures, standardized assessments, and teacher-centered instruction, are increasingly being challenged by digital innovations that enable more dynamic, interactive, and personalized learning experiences. Among these innovations, artificial intelligence (AI) technologies occupy a central role, driving a paradigm shift in how knowledge is delivered, acquired, and assessed. AI systems can analyze vast amounts of educational data, identify learning patterns, and adapt content to individual student needs, thereby introducing innovative, interactive, and adaptive dimensions to teaching and learning processes (Martínez-Hernández, 2025; Moroianu et al., 2023).

These technologies are particularly relevant in the disciplines of Science, Technology, Engineering, and Mathematics (STEM), which are characterized by abstract concepts, complex problem-solving tasks, and rapidly evolving knowledge domains (L. Chen et al., 2020). AI-powered simulations enable learners to visualize abstract scientific phenomena that are otherwise difficult to observe directly, such as molecular interactions, physical forces, or dynamic systems, thereby supporting conceptual understanding in science education (de Jong & van Joolingen, 1998; Rutten et al., 2012). Similarly, adaptive learning platforms and intelligent tutoring systems dynamically adjust the difficulty, sequencing, and feedback for engineering and mathematics tasks based on learners’ real-time performance, ensuring that instruction remains both appropriately challenging and attainable (VanLehn, 2011; Aleven et al., 2016). Beyond individual learning processes, the integration of AI into educational management and learning analytics systems has been shown to enhance decision-making related to scheduling, resource allocation, and formative assessment by leveraging large-scale student data (Siemens & Long, 2011; Romero & Ventura, 2020). Such systems support instructors and administrators in monitoring learning progress, identifying at-risk students, and optimizing instructional interventions in a timely manner. Collectively, these applications demonstrate that AI not only facilitates the adoption of innovative instructional strategies but also improves the quality of interaction, engagement, and personalized support among students, teachers, and educational institutions (OECD, 2021).

STEM education plays a critical role in preparing learners for the knowledge-based society of the 21st century, equipping them with essential competencies such as critical thinking, computational reasoning, and collaborative problem-solving skills that are increasingly demanded in modern workplaces (Nadelson & Seifert, 2017; Cao, 2025). Despite its centrality to future workforce development, STEM education faces persistent and multifaceted challenges. Abstract and conceptually complex topics are often difficult to convey through conventional instruction alone, while opportunities for practical, hands-on, and experiential learning are frequently constrained by limited resources, time, or teacher expertise (Hayes & Kraemer, 2017; Thomas & Watters, 2015; J. Huang et al., 2025). In addition, students’ interest and motivation in STEM disciplines can decline due to systemic inequalities, gender disparities, and the perceived difficulty of the subject matter. Traditional instructional models, many of which were developed in the early 20th century, are frequently inadequate to meet these challenges, highlighting the urgent need for innovative, technology-enhanced solutions that can bridge the gap between theoretical knowledge and practical application, engage diverse learners, and foster the development of 21st-century skills.

## 2. Literature Review

### 2.1. AI in STEM Education

Artificial intelligence (AI) has emerged as a transformative force in education, offering solutions to many of the persistent challenges faced in STEM learning. AI-supported educational tools, including intelligent tutoring systems, adaptive learning platforms, chatbots, virtual laboratories, and interactive simulations, provide new ways to deliver content, monitor student progress, and facilitate skill development. These tools enable personalized learning experiences, provide real-time feedback on performance, and help visualize complex or abstract concepts that are often difficult to grasp through conventional instruction alone (García-Martínez et al., 2023; Hu, 2024). For instance, virtual laboratories enable students to conduct experiments in safe, controlled, and repeatable environments, allowing them to explore scientific phenomena without the constraints of physical resources or safety risks, which has been shown to enhance conceptual understanding and inquiry skills (de Jong et al., 2013; Makransky et al., 2019). Likewise, adaptive learning platforms dynamically adjust the difficulty level, sequencing, and pacing of instructional tasks based on learners’ ongoing performance, thereby maintaining an optimal level of cognitive engagement and supporting more effective learning processes (Aleven et al., 2016; Kovanović et al., 2015).

Popenici and Kerr (2017) define AI as information-processing systems capable of executing human-like cognitive processes such as learning, adaptation, synthesis, and self-correction. In the educational context, this translates to systems that can provide individualized guidance, scaffolding, and feedback, enabling students to learn at their own pace and according to their preferred learning styles. Such personalization has been shown to improve comprehension, retention, and mastery of challenging STEM topics, bridging gaps that conventional one-size-fits-all instruction often cannot address.

Empirical research indicates that integrating AI technologies into classroom environments positively affects student achievement and learning outcomes (Harry & Sayudin, 2023). By fostering interactive, adaptive, and responsive learning experiences, AI tools complement traditional teaching methods and facilitate the translation of theoretical knowledge into practical understanding (García-Martínez et al., 2023; Hu, 2024). Furthermore, AI applications have the potential to enhance student motivation, engagement, and attitudes toward STEM disciplines, which are factors critical for sustained interest and long-term academic success (Dong et al., 2025). For example, students who engage with AI-supported simulations frequently report increased confidence and self-efficacy in addressing complex scientific and mathematical problems, as these environments allow for iterative exploration and immediate feedback (Sitzmann, 2011). Similarly, intelligent tutoring systems (ITS) have been shown to reduce learners’ frustration and anxiety by providing timely hints, step-by-step guidance, and adaptive scaffolding, thereby supporting persistence and emotional regulation during problem-solving tasks (Aleven et al., 2016; D’Mello & Graesser, 2012).

Beyond individual learning, AI also holds substantial promise for educators and institutions by enabling data-driven instructional decision-making. Through learning analytics and AI-powered dashboards, teachers can monitor student performance in real time, identify misconceptions and knowledge gaps, and adapt instruction accordingly, supporting more targeted and effective pedagogical interventions (Holstein et al., 2019; Romero & Ventura, 2020). At the institutional level, AI-driven analytics provide evidence to inform curriculum design, instructional sequencing, and assessment practices, allowing decision-makers to identify which teaching strategies and digital tools yield the most effective learning outcomes in STEM education (Siemens & Baker, 2012; Ferguson, 2012). In this way, AI supports not only classroom-level instructional improvement but also broader curriculum development and educational policy grounded in empirical learning data (X. Chen et al., 2020).

Overall, the integration of AI into STEM education represents not just a technological innovation but a pedagogical evolution. By addressing both cognitive and motivational challenges, AI provides scalable solutions that can transform the teaching and learning landscape, helping students acquire the critical skills required for success in the 21st-century knowledge economy.

### 2.2. Evidence from Empirical Studies

In recent years, there has been a significant increase in experimental and quasi-experimental research investigating the effects of AI-supported instructional practices across different countries, educational levels, and STEM disciplines. Accumulating empirical evidence suggests that AI-based interventions—such as intelligent tutoring systems, adaptive learning environments, and data-driven feedback tools—can produce meaningful improvements in student learning outcomes, particularly in addressing the conceptual complexity and individual learning differences characteristic of STEM education (L. Chen et al., 2020; García-Martínez et al., 2023). These studies collectively indicate that AI interventions can have substantial benefits for student learning, particularly in addressing the unique challenges posed by STEM education. By providing personalized guidance, interactive simulations, and adaptive feedback, AI tools enable students to engage with complex concepts more effectively and at their own pace (García-Martínez et al., 2023; Hu, 2024).

Meta-analytic and systematic review studies offer robust evidence for the positive impact of AI on learning outcomes (Ouyang et al., 2023; Ouyang & Xu, 2024; Taheri et al., 2025; Trapero-González et al., 2025; Xu & Ouyang, 2022; Younas et al., 2025). For instance, Dong et al. (2025) conducted a meta-analysis that revealed AI-assisted teaching significantly enhances students’ academic achievement, demonstrating a clear advantage over traditional instruction in various STEM contexts. Similarly, García-Martínez et al. (2023) found that AI and computational science applications exert a significantly positive influence on student performance, particularly within STEM knowledge domains. Hu (2024), through a meta-analysis of 31 empirical studies, reported that AI-supported personalized learning produces moderately positive effects not only on cognitive outcomes, such as knowledge acquisition and skill development, but also on emotional and affective aspects, including student motivation and self-efficacy.

Despite these promising results, empirical findings are not fully consistent. Variability in research designs, sample characteristics, types of AI tools employed, and contextual factors such as curriculum integration and classroom practices contribute to differing outcomes across studies (Hwang et al., 2020a). Some research emphasizes the transformative potential of AI, positioning it as a tool capable of fundamentally improving traditional educational methods. Conversely, other studies caution that AI’s effectiveness may be contingent upon factors such as student demographics, instructional design, and the duration or intensity of the intervention (Dong et al., 2025).

Importantly, a growing body of research also highlights potential limitations and negative effects associated with AI-supported instruction, underscoring the need for a balanced evaluation. For instance, some studies have shown that technology-enhanced environments, including immersive and AI-supported simulations, may increase cognitive load when instructional design is not aligned with learners’ prior knowledge, potentially resulting in reduced learning outcomes (Makransky et al., 2019). In addition, scholars have cautioned that excessive reliance on AI-generated feedback and automated guidance may lead to superficial engagement, reduced learner autonomy, and overdependence on system support, particularly among less experienced learners (Holmes et al., 2019; Luckin et al., 2016). Recent research on generative AI further raises concerns regarding inaccuracies, hallucinated content, and the risk that students may adopt incorrect or unverified information without sufficient critical evaluation (Yang et al., 2023). Moreover, the benefits of AI-supported learning are not distributed evenly across learners; differences in digital literacy, access to technology, and self-regulation skills may exacerbate existing educational inequalities (Kohnke & Zaugg, 2025). Taken together, these findings suggest that the effectiveness of AI in education is not universally positive but is highly contingent upon instructional design quality, learner characteristics, and contextual implementation factors.

These inconsistencies highlight the need for systematic synthesis and quantitative assessment of existing research to determine the overall magnitude of AI’s effects in STEM education. By aggregating findings across studies and accounting for moderating factors, meta-analytic approaches can provide clearer evidence regarding the effectiveness of AI interventions, inform best practices, and guide the development of policies and curricula that optimize learning outcomes in STEM disciplines.

### 2.3. Need for Meta-Analytic Synthesis and Research Objectives

Meta-analysis offers a powerful approach for aggregating results from independent studies, providing more robust estimates of overall effect sizes and the influence of moderating variables such as educational level, STEM discipline, and intervention duration (Borenstein et al., 2009). Synthesizing existing evidence is particularly valuable for rapidly evolving technologies like AI, whose integration into education is increasing but for which empirical findings remain heterogeneous (Crompton & Burke, 2023).

Despite the growing body of research on AI-supported STEM education, the findings are often inconsistent due to differences in research designs, sample characteristics, types of AI tools, and implementation contexts (Hwang et al., 2020b). This highlights the need for a systematic and quantitative synthesis to identify the overall magnitude of AI’s effect on student learning outcomes and to examine the factors that may moderate this effect.

To address these gaps, the current study conducts a meta-analysis of experimental and quasi-experimental research on AI-supported STEM education. The primary objectives of this study are as follows:

1. To quantify the overall effect of AI interventions on student academic achievement, operationalized primarily as performance on achievement tests, examinations, and standardized assessments.
2. To examine the moderating effects of educational level, STEM discipline, and intervention duration on learning achievement.
3. To provide evidence-based insights for policymakers, curriculum developers, and teachers to guide the effective use of AI technologies in educational contexts.

Accordingly, this study is guided by the following research questions:

1. Overall Effectiveness: What is the overall effect of AI-supported instructional interventions on student academic achievement—as measured by achievement tests, examinations, and standardized assessments—in STEM education compared to traditional teaching methods?
2. Educational Level as a Moderator: How does the effectiveness of AI-supported instruction vary across different educational levels (e.g., primary, secondary, and tertiary education)?
3. STEM Discipline as a Moderator: To what extent does the impact of AI-supported interventions differ across STEM disciplines, such as Science, Technology/Engineering, and Mathematics?
4. Intervention Duration as a Moderator: How does the duration of AI-supported interventions influence their effectiveness in improving learning outcomes?
5. Implications for Practice and Policy: What evidence-based insights can be derived from existing research to guide the effective integration of AI technologies in STEM classrooms, curriculum design, and educational policy?

By systematically synthesizing current research and quantifying effect sizes, this study not only contributes to the growing literature on AI in STEM education but also identifies critical directions for future research and areas where practical innovations are most needed.

## 3. Method

This meta-analysis study was conducted in accordance with the PRISMA guidelines (Page et al., 2021) to obtain systematic and reliable answers to the research questions (Supplementary Material). The review protocol was registered on OSF (Registration DOI: https://doi.org/10.17605/OSF.IO/BQZ7W (accessed on 10 June 2026)). The main objective of the study is to systematically synthesize the findings of experimental research examining the effects of AI applications on learning achievement in STEM fields. Accordingly, a comprehensive literature review was carried out. The databases to be used in the search, as well as the inclusion and exclusion criteria for the studies, were determined in advance. The databases examined include Web of Science, Scopus, ERIC, ScienceDirect, Google Scholar, and the Council of Higher Education National Thesis Center.

To identify studies for inclusion in the research, a systematic literature search was conducted in both Turkish and English. During this process, search strings were structured using Boolean logic (AND, OR, NOT operators). The operator “OR” was used between keywords, and an asterisk (*) was added to the end of certain words to include their various forms. In addition, quotation marks (“ ”) were used to search for specific terms in their exact form. The keywords were organized under two main thematic categories aligned with the research objectives:

- Terms related to artificial intelligence: “yapay zekâ,” “artificial intelligence,” “AI,” “makine öğrenmesi,” “machine learning,” “derin öğrenme,” “deep learning,” “chatbot,” “akıllı öğretim sistemi,” “intelligent tutoring system,” “uyarlanabilir öğrenme,” “adaptive learning,” “eğitim teknolojisi,” “educational technology”;
- Terms related to STEM and learning achievement: “STEM eğitimi,” “STEM education,” “fen eğitimi,” “science education,” “teknoloji eğitimi,” “technology education,” “mühendislik eğitimi,” “engineering education,” “matematik eğitimi,” “mathematics education,” “öğrenme başarısı,” “learning achievement,” “akademik performans,” “academic performance,” “öğrenci başarısı,” “student success,” “deneysel çalışma,” “experimental study.”

A systematic approach was adopted in the selection of keywords. Searches were conducted across the title, abstract, and keyword fields of each database. An example search string applied in Web of Science was as follows: (“artificial intelligence” OR “AI” OR “machine learning” OR “deep learning” OR “chatbot” OR “intelligent tutoring system” OR “adaptive learning” OR “educational technology”) AND (“STEM education” OR “science education” OR “technology education” OR “engineering education” OR “mathematics education”) AND (“learning achievement” OR “academic performance” OR “student success” OR “experimental study”). Search strings were adapted to the syntax requirements of each database while maintaining conceptual consistency across all platforms.

The literature searches were conducted in September 2025 through the databases identified earlier. All studies published on the topic were initially considered within the scope of the review. However, after duplicate records were removed, only the studies meeting the following inclusion criteria were selected for meta-analysis:

- Inclusion of AI-supported applications in STEM fields;
- Employment of an experimental or quasi-experimental research design;
- Reporting of quantitative data related to learning achievement.

Studies that did not meet the specified inclusion criteria, did not provide sufficient statistical data, or lacked an experimental design were excluded from the analysis. The lower boundary of January 2005 was established to ensure a comprehensive search window that captured the early development of AI-supported instructional tools in STEM education, as foundational meta-analytic work on intelligent tutoring systems dates back to this period (Ma et al., 2014; Kulik & Fletcher, 2016). Although the majority of included studies (*n* = 29) were published after 2020, reflecting the accelerated adoption of AI technologies in education, the broader timeframe ensured that no relevant early empirical work was excluded from consideration. The inclusion and exclusion criteria used to determine the studies incorporated into the meta-analysis are presented in detail in Table 1.

### 3.1. Data Extraction and Quality Assessment

**Figure 1:**
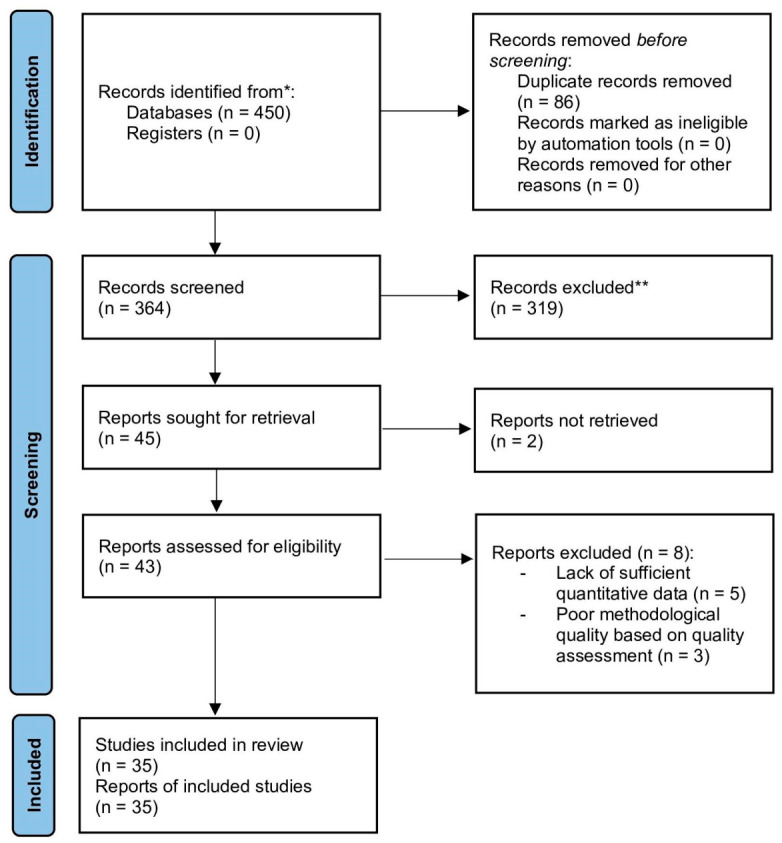
Flow chart for selection of works (flow diagram). * Databases searched: Scopus, Web of Science, and ERIC. No records were identified from registers. ** Records excluded after title and abstract screening because they did not meet the inclusion criteria.

All studies were independently reviewed and evaluated by the researchers. The data extraction process was visualized using the PRISMA flow diagram (Figure 1). This process was carried out in three stages.

The study selection process followed the PRISMA 2020 Statement guidelines (Page et al., 2021). A total of 450 records were identified through database searching, with no additional records identified from registers. Before screening, 86 duplicate records were removed, and no records were excluded by automation tools or for other reasons. Following the removal process, 364 records were screened based on titles and abstracts, of which 319 were excluded. Subsequently, 45 reports were sought for retrieval, and 2 reports could not be retrieved. A total of 43 full-text reports were assessed for eligibility. Of these, 8 reports were excluded due to a lack of sufficient quantitative data (*n* = 5) and poor methodological quality based on the quality assessment (*n* = 3). Finally, 35 studies met the inclusion criteria and were included in the systematic review, with 35 corresponding reports.

These data not only enabled the compilation of key statistical information required for effect size calculations but also allowed for the assessment of the contextual characteristics of the studies. Moreover, this information served as an important resource for forming subgroups used in the moderator analyses. Rather than applying a formal quality appraisal tool, methodological quality was operationalized through predefined inclusion and exclusion criteria (see Table 1), which required studies to employ experimental or quasi-experimental designs and to report sufficient statistical data for effect size calculation. This approach ensured a minimum threshold of methodological rigor for all included studies.

Basic information regarding the studies included in the meta-analysis is systematically presented in Appendix A. These references contribute to the transparent reporting of the primary studies used in the analysis and enhance the traceability of the research process.

### 3.2. Coding of Studies

To ensure standardized data collection for the studies included in this meta-analysis, a comprehensive coding form was developed. This coding form enabled the systematic classification of information provided by the studies and its preparation for analysis. The following categories were taken into account during the coding process:

- Author(s): The author(s) of the study were recorded.
- Year of Study: The year of publication was noted.
- Publication Type: The type of publication (article, thesis, or conference paper) was specified.
- Educational Level/Class: The educational level at which the research was conducted (primary, secondary, high school, or university) was recorded.
- Subject Area: The discipline in which the study was conducted (Science, Technology/Engineering, Mathematics) was specified.
- Duration of Intervention: The duration of the AI-based intervention was recorded (≤5 h; >1, ≤7 days; >1, ≤4 weeks; >1, ≤2 months; >2, ≤3 months; >3 months).
- Class Size: The number of students in the sample was categorized as small (≤49 students), medium (50–99 students), or large (≥100 students).
- Experimental/Control Group Data: The mean (M), standard deviation (SD), and sample size (N) for both experimental and control groups were extracted.

The coding process was conducted by three researchers. To ensure consistency and reliability, the coders first worked simultaneously on pilot studies identified in the preliminary phase and standardized the coding form. This stage was critical for testing the applicability of the form and for developing a shared understanding among the coders. Thus, potential inconsistencies and ambiguities were resolved before proceeding to the broader dataset. Subsequently, the studies were randomly assigned to two coders for independent coding. In total, 35 studies were coded, and inter-rater reliability was calculated using Cohen’s Kappa statistic, yielding a coefficient of κ = 0.93, which indicates a high level of agreement.

In this study, statistically independent effect sizes were used to ensure the validity of the meta-analytic findings (Scammacca et al., 2014). Dependency in a meta-analysis can arise when studies use multiple outcome measures on the same participants or when two intervention groups are compared against the same control group. To avoid potential dependent effect sizes, only one effect size was selected from each included study. When multiple eligible outcomes were reported, the primary outcome measure most closely aligned with overall academic achievement was preferred. When calculating the effect sizes from the 35 included studies, Hedges’ g was preferred over Cohen’s d. Cohen’s d tends to bias the estimation of population effect sizes in studies with small sample sizes. Effect sizes were calculated using the post-test means, standard deviations, and sample sizes of the experimental and control groups. In contrast, Hedges’ g applies a correction factor to reduce this bias. The correction formula used for Hedges’ g is provided below (Borenstein et al., 2009):

$$Hedges\text{’}g=Cohen\text{’}s\;d\;\times \;\left(1-\frac{3}{4\;\times \;\left(N_{exp}+N_{cont}\right)-9}\right)$$

$\frac{}{4\;\times \;\left(\right)-9}$ This correction ensures that Hedges’ g provides a more appropriate and reliable effect size estimate for the present study. To synthesize the impact of AI interventions on students’ academic achievement, the Random Effects Model (REM) was employed as the analytical framework. The choice of REM over the Fixed-Effect Model (FEM) is justified by the need to account for heterogeneity among studies, which was assessed using the Q test, while inconsistencies across study results were quantified using the I^2^ statistic, and the between-study variance (τ^2^) was estimated. Forest plots were used to visualize the effect sizes and their confidence intervals. Additionally, various moderator analyses were conducted to examine the influence of variables such as educational level, intervention duration, sample size, and subject area on the effectiveness of AI interventions. All analyses were performed using Comprehensive Meta-Analysis (CMA) 3.0 software.

## 4. Results

An examination of Table 2 reveals that the results obtained under both the FEM and the REM indicate statistically significant and positive effect sizes. The FEM analysis, which includes 35 studies, demonstrates a statistically significant effect (g = 0.593, 95% CI [0.522, 0.664]) with Z = 16.36 and *p* < 0.001. The meta-analytic findings indicate that there is a significant and high level of heterogeneity among the included studies (Q = 199.76, I^2^ = 82.98%). This finding suggests that considerable variation exists across studies and that the effect sizes do not display a homogeneous distribution.

The REM analysis presented in Table 2 takes into account heterogeneity among studies, allowing for more reliable and generalizable estimates of effect sizes. Under this model, the effect size was found to be g = 0.670 (95% CI [0.491, 0.848]), with Z = 7.35 and *p* < 0.001, while the between-study variance was accounted for more appropriately under the REM (I^2^ = 15.75%). This indicates that the REM, by incorporating between-study variance into its estimations, provides a more conservative and generalizable effect-size estimate compared to the FEM.

In conclusion, the findings presented in Table 2 show that the analyzed intervention generally produces a moderate and positive effect on learning outcomes. Considering the observed heterogeneity among studies, it is concluded that models accounting for heterogeneity, such as the REM, are more appropriate for analysis.

The moderator analyses reported in Table 3 indicate that the effect sizes differ significantly across various groups. Based on student level, the highest effect size was observed at the high school level (g = 1.099, 95% CI [0.89, 1.30], Z = 10.53, *p* < 0.001). In contrast, the effects were lower at the elementary school (g = 0.465) and middle school (g = 0.392) levels. For university students, a moderate effect was identified (g = 0.578, 95% CI [0.48, 0.68], Z = 11.67, *p* < 0.001). The test of between-group heterogeneity was significant (Q = 30.13, df = 3, *p* < 0.001).

Regarding intervention duration, the highest effect size was observed in interventions lasting more than 1 month and up to 2 months (g = 0.833, 95% CI [0.67, 0.99], Z = 10.30, *p* < 0.001). Short-term interventions (≤5 h) also showed a significant effect (g = 0.621, *p* = 0.003). However, for interventions lasting more than 1 and up to 7 days, the effect was weaker and not statistically significant (g = 0.256, *p* = 0.070). The between-group heterogeneity was significant (Q = 16.95, df = 5, *p* = 0.004).

In terms of subject area, the highest effects were observed in science (g = 0.676) and mathematics (g = 0.650). In technology and engineering courses, the effect size was slightly lower (g = 0.501). However, the between-group heterogeneity was not statistically significant (Q = 4.85, df = 2, *p* = 0.088), indicating that the differences in effect sizes across STEM disciplines should be interpreted with caution.

Analyses based on class size revealed that the effect size was highest in medium-sized classes (g = 0.687). In small classes, the effect was lower (g = 0.448), while in large classes, it was moderate (g = 0.545). The between-group heterogeneity was marginally significant (Q = 4.61, df = 2, *p* = 0.099).

Overall, the findings show that the total effect size across all moderators was g = 0.593 (95% CI [0.52, 0.66], Z = 16.47, *p* < 0.001), indicating a moderate and positive effect of AI-based interventions on students’ academic achievement.

**Figure 2:**
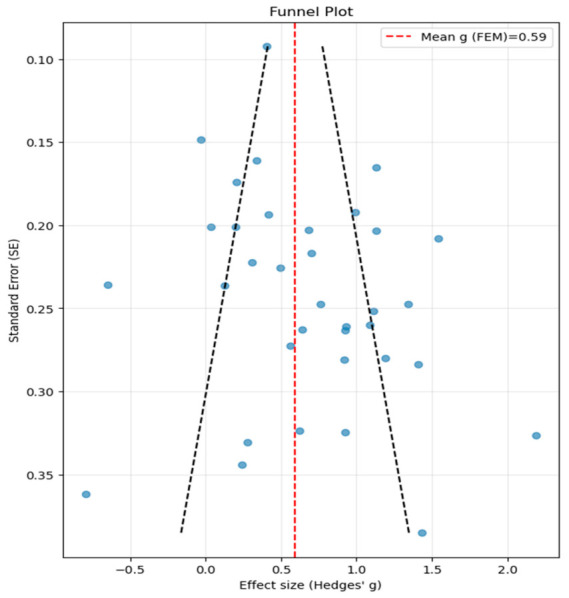
Funnel plot.

The funnel plot presented in Figure 2 illustrates the distribution of studies’ effect sizes (Hedges’ g) and their standard errors (SE). The dots in the graph represent individual studies included in the meta-analysis. When the dots are symmetrically distributed, it suggests a low likelihood of publication bias.

An examination of Figure 2 reveals that the funnel plot demonstrates a generally symmetrical distribution. The funnel plot demonstrates a generally symmetrical distribution around the mean effect size. A small number of studies appear as outliers on the right side of the plot, reflecting higher effect sizes. Overall, the funnel plot does not display significant asymmetry, suggesting no strong evidence of publication bias.

Nevertheless, since visual inspection alone is insufficient for a definitive conclusion, the potential for publication bias was further tested statistically using Begg’s and Egger’s regression analyses, and the corresponding results are reported in Table 4.

The analyses conducted to assess publication bias (Table 4) revealed that the Egger’s regression test statistic was not significant (*p* = 0.095). This finding indicates that there is no strong evidence of systematic asymmetry or publication bias in the meta-analysis. Similarly, the Begg’s test yielded a tau value of −0.2303 with a corresponding *p*-value of 0.0517, which, while suggesting a marginal indication of possible publication bias, is not statistically significant (*p* > 0.05).

Following adjustment using the Trim-and-Fill method, the original mean effect size (g_mean = 0.6799) was recalculated as 0.7269, representing a slight increase. The adjusted confidence interval was estimated as [0.6696, 0.7841], and only one missing study was imputed. It is worth noting that the Trim-and-Fill adjustment resulted in a slight increase in the effect size rather than a decrease, which may reflect sampling variability, given that only one missing study was imputed. These results suggest that publication bias, if present, did not substantially alter the overall conclusions of the meta-analysis. Nevertheless, given the borderline Begg’s test result (*p* = 0.0517), these findings should be interpreted with appropriate caution.

In addition, Rosenthal’s fail-safe number analysis was performed to further evaluate the robustness of the meta-analytic results against publication bias. The corresponding results are presented in Table 5.

An examination of Table 5 reveals that the combined effect size obtained from the 35 studies included in the meta-analysis is statistically significant (Z = 16.36, *p* < .001). The Z-value being well above the critical threshold of 1.96 indicates that the observed effect is not due to random variation and is statistically significant at a high level. This finding provides strong meta-analytic support for the relationship or intervention effect examined across studies.

Additionally, a two-tailed test approach was used in the analysis, which implies that no prior assumption was made about the direction of the effect and that the possibility of significance in both directions was considered.

Furthermore, the Fail-safe N value was calculated as 2404, meaning that 2404 additional “null-effect” studies (i.e., studies with zero effect size) would need to be added to the analysis to render the overall result non-significant. According to Rosenthal’s (1979) criteria, such a high Fail-safe N value indicates that the meta-analysis results are robust and that potential publication bias does not pose a serious threat to the validity of the findings.

In conclusion, the meta-analysis results demonstrate a strong and reliable overall effect within the examined research domain. When considered together, the significant Z-value, low *p*-value, and high Fail-safe N confirm that the findings are both statistically and methodologically consistent and trustworthy.

## 5. Discussion

### 5.1. Interpretation of the Overall Effect

The present meta-analysis demonstrates a moderate to strong positive effect of AI-supported instruction on student achievement in STEM education (Hedges’ g ≈ 0.67, *p* < .001). This finding provides robust quantitative evidence that AI-based instructional interventions can meaningfully enhance learning outcomes across diverse educational contexts. The magnitude of this effect is educationally meaningful and aligns with a growing body of research suggesting that AI-driven systems can support personalized learning pathways, adaptive feedback, and data-informed instructional decision-making (Holmes et al., 2019; Luckin et al., 2016).

However, the observed effect size should not be interpreted as evidence of universally transformative impact. Rather, it reflects a conditional and context-sensitive effectiveness, shaped by variations in instructional design, implementation fidelity, and learner characteristics. The substantial heterogeneity observed across studies (I^2^ ≈ 83%) indicates that AI-supported instruction does not yield uniform outcomes and that its effectiveness is mediated by multiple interacting variables (Hwang et al., 2020a). This variability suggests that AI is not inherently effective, but becomes effective under specific pedagogical and contextual conditions.

From a theoretical standpoint, these findings can be interpreted through several complementary learning frameworks. First, cognitive load theory offers a compelling explanation for the observed effects. AI-supported systems have the potential to optimize cognitive processing by structuring content, sequencing tasks, and providing immediate feedback that reduces extraneous cognitive load while supporting germane processing (Sweller, 2011). At the same time, the presence of heterogeneous outcomes suggests that not all implementations achieve this balance; poorly designed AI environments may increase cognitive demands, particularly when learners lack sufficient prior knowledge or when interfaces are overly complex (Makransky et al., 2019).

Second, from a constructivist and socio-cultural perspective, AI tools can be conceptualized as mediational artifacts that support active knowledge construction through interaction, feedback, and iterative engagement (Bruner, 1996). In particular, intelligent tutoring systems and generative AI applications function as adaptive scaffolds aligned with Vygotsky’s (1978) zone of proximal development, providing just-in-time support tailored to learners’ evolving competencies. Importantly, this perspective highlights that learning gains are not solely a product of technology, but of the interaction between learner, tool, and instructional context.

Third, the findings can be situated within broader educational technology integration frameworks, such as TPACK and SAMR (Mishra & Koehler, 2006; Puentedura, 2013). These frameworks emphasize that the educational value of technology depends on its alignment with pedagogical and content knowledge. The moderate effect size observed in this study suggests that AI-supported instruction most often enhances or modifies existing teaching practices rather than fundamentally transforming them. This reinforces the view that AI functions primarily as an augmentative technology, amplifying effective pedagogy rather than substituting for it (Holmes et al., 2019).

At a broader level, the findings also resonate with emerging perspectives on human–AI complementarity in education, which emphasize that optimal learning outcomes arise when AI systems and human teachers perform complementary roles. While AI can provide scalable personalization and real-time feedback, teachers contribute critical elements such as pedagogical judgment, socio-emotional support, and ethical oversight. The effectiveness observed in this meta-analysis likely reflects the degree to which these complementary roles are successfully integrated within instructional environments.

### 5.2. Moderating Factors and Contextual Variability

The moderator analyses provide further insight into the contextual conditions that shape the effectiveness of AI-supported instruction. Rather than producing uniform benefits, AI interventions appear to operate differently across educational levels, durations, and instructional contexts.

The finding that the strongest effects occur at the high school level suggests that learner readiness is a key moderating factor. High school students typically possess more developed cognitive and metacognitive capacities, enabling them to engage more effectively with adaptive feedback, self-paced learning, and complex digital interfaces (Zimmerman, 2002). In contrast, younger learners may require more structured guidance and teacher mediation, which may limit the effectiveness of AI systems when implemented without sufficient scaffolding.

The role of intervention duration further highlights the importance of temporal dynamics in learning with AI. The finding that interventions lasting between one and two months produce the strongest effects suggests that learning gains emerge through sustained interaction rather than immediate exposure. This aligns with theories of skill acquisition and self-regulated learning, which emphasize the importance of repeated practice, feedback cycles, and gradual internalization of strategies (Ma et al., 2014). At the same time, the absence of a strictly increasing pattern with longer durations indicates that more time does not automatically translate into greater effectiveness, pointing to the importance of instructional quality over mere exposure.

Although disciplinary differences were not statistically significant, the slightly higher effects observed in science and mathematics may reflect the alignment between AI capabilities and the structured, problem-based nature of these domains (Kulik & Fletcher, 2016). However, the lack of significant differences suggests that AI-supported instruction has cross-disciplinary potential, provided that tools are appropriately adapted to domain-specific learning processes.

Taken together, these findings reinforce the conclusion that AI-supported instruction is not a one-size-fits-all solution, but a context-dependent intervention whose effectiveness emerges from the alignment of learner characteristics, instructional design, and implementation conditions.

### 5.3. Educational Implications

The findings of this meta-analysis have several important implications for educational practice, particularly in relation to how AI technologies should be integrated into teaching and learning environments.

First, AI should be conceptualized as a pedagogical partner rather than an autonomous instructional agent. The results clearly indicate that AI does not replace the role of the teacher but instead extends instructional capacity. Teachers remain essential for guiding interpretation, facilitating deeper understanding, and supporting higher-order thinking processes (Luckin et al., 2016; Holmes et al., 2019).

Second, the effectiveness of AI-supported instruction is highly dependent on instructional design quality. AI tools must be carefully aligned with learning objectives, structured to manage cognitive load, and embedded within coherent pedagogical strategies. Poorly designed implementations risk promoting superficial engagement, over-reliance on automated feedback, or cognitive overload.

Third, the findings highlight the growing importance of AI literacy and pedagogical competence among educators. Teachers must be equipped not only to use AI tools but also to critically evaluate their outputs, understand their limitations, and integrate them meaningfully into instructional practice (Holstein et al., 2019). This includes supporting students in developing critical engagement with AI-generated content, particularly in the context of generative AI.

Fourth, the integration of AI in education raises important issues related to equity, access, and ethical use. Differences in digital infrastructure, learner readiness, and access to resources may influence who benefits most from AI-supported instruction (Kohnke & Zaugg, 2025). Without careful implementation, AI may risk reinforcing existing educational inequalities rather than reducing them.

Finally, the findings suggest that effective AI integration requires system-level planning and sustained implementation, rather than short-term or isolated interventions. Educational institutions should prioritize long-term integration strategies, continuous evaluation, and alignment with curricular and pedagogical goals.

### 5.4. Limitations and Future Research

Despite the strengths of this meta-analysis, several limitations should be acknowledged. The high level of heterogeneity indicates that a substantial proportion of variance remains unexplained, suggesting the need for future research to examine additional moderating variables, including implementation fidelity, teacher involvement, and learner characteristics (Hwang et al., 2020a).

Moreover, the predominance of short- to medium-term interventions limits our understanding of the long-term sustainability of AI-supported learning gains. Longitudinal studies are needed to examine whether these effects persist over time and transfer to broader learning contexts (Ma et al., 2014).

Future research should also move beyond cognitive outcomes to include affective, motivational, and metacognitive dimensions of learning, which are increasingly recognized as critical in AI-supported environments. In addition, more research is needed on the role of generative AI, particularly in relation to issues of accuracy, trust, and critical thinking.

Finally, cross-cultural and contextual differences remain underexplored. Comparative research examining how institutional, cultural, and technological factors influence the effectiveness of AI-supported instruction would provide valuable insights for both research and practice.

## 6. Conclusions

In conclusion, this meta-analysis provides strong evidence that AI-supported instruction can enhance student achievement in STEM education, yielding moderate to strong positive effects. However, these effects are not uniform and are shaped by a complex interplay of pedagogical, technological, and learner-related factors.

AI appears to be most effective when implemented within well-designed instructional environments, supported by teacher expertise, and aligned with learners’ developmental readiness. Rather than serving as a replacement for traditional instruction, AI functions most effectively as a complementary tool that enhances and extends pedagogical practice.

Looking ahead, the successful integration of AI in education will depend on the ability to balance technological innovation with pedagogical integrity, ethical considerations, and sustained implementation. When these elements are aligned, AI has the potential to support more personalized, adaptive, and effective learning experiences in STEM education.

## Figures and Tables

**Table 1:** Inclusion and exclusion criteria.

Criterion	Inclusion Criteria	Exclusion Criteria
Learning Approach	Studies must involve instructional practices in STEM fields that utilize AI technologies or include AI-supported interventions (e.g., machine learning, chatbots, intelligent tutoring systems, etc.).	Studies that do not use AI technology and are conducted solely through traditional teaching methods were excluded.
Publication Date	Studies published between 1 January 2005 and 1 September 2025 were included.	Studies published outside this time frame were excluded from the analysis.
Publication Type	Priority was given to studies published in peer-reviewed journals. However, preprints on arXiv, postgraduate theses, and conference papers were also considered if they met the necessary criteria.	Publications such as book chapters and letters to the editor were not included.
Language	Studies must be written in Turkish or English.	Studies published in languages other than Turkish or English were excluded.
Research Design	Studies must employ an experimental or quasi-experimental design. They should include experimental and control groups, where the experimental group receives an AI-based intervention and the control group follows traditional instruction.	Studies that do not use experimental or quasi-experimental designs, lack pre-test–post-test data, or do not include comparative groups were excluded.
Educational Outcomes	Studies must report measurable outcomes related to learning achievement or academic performance (e.g., exam scores, achievement tests, knowledge scores).	Studies lacking academic achievement measures or relying solely on subjective evaluations were excluded.
Research Results	Included studies must provide sufficient statistical data for both experimental and control groups (N, mean [M], standard deviation [SD]) and include the information necessary to calculate effect size.	Studies that otherwise met the inclusion criteria but did not provide sufficient statistical data for effect size calculation were excluded.

**Table 2:** Meta-analysis results.

Model Type	*n*	Z	*p*	Q	g	I^2^	95% Confidence Interval	
							Lower Limit	Upper Limit
FEM	35	16.3602	0.000	199.762	0.5931	82.98	0.5220	0.6641
REM	35	7.3539	0.000	40.355	0.6696	15.75	0.4912	0.8481

*df*: 34.

**Table 3:** Moderator analyses.

Moderator	Groups	Effect Size and 95% Confidence Interval				Test of Null		Heterogeneity		
		*n*	g	Lower	Upper	Z-Value	*p*-Value	Q-Value	df	*p*-Value
Students’ Level	Primary	4	0.465	0.31	0.62	6.04	0.000	30.13	3	0.000
	Middle	5	0.392	0.19	0.60	3.79	0.002			
	High School	5	1.099	0.89	1.30	10.53	0.000			
	University	21	0.578	0.48	0.68	11.67	0.000			
	Tot. Betw. Overall	35	0.593	0.52	0.66	16.47	0.000			
Durations of Implementation	≤5 h	2	0.621	0.28	0.96	3.59	0.003	16.95	5	0.004
	>1, ≤7 days	3	0.256	0.02	0.53	1.81	0.070			
	>1, ≤4 weeks	7	0.456	0.28	0.63	5.12	0.000			
	>1, ≤2 months	10	0.833	0.67	0.99	10.30	0.000			
	>2, ≤3 months	3	0.618	0.38	0.86	5.04	0.000			
	>3 months	10	0.576	0.47	0.68	10.52	0.000			
	Tot. Betw. Overall	35	0.593	0.52	0.66	16.47	0.000			
School Subjects	Maths	10	0.650	0.53	0.77	10.47	0.000	4.85	2	0.088
	Science	9	0.676	0.53	0.82	9.03	0.000			
	Technology/Engineering	16	0.501	0.39	0.61	9.02	0.000			
	Tot. Betw. Overall	35	0.593	0.52	0.66	16.47	0.000			
Class Sizes	Small	6	0.448	0.17	0.72	3.20	0.000	4.61	2	0.099
	Medium	18	0.687	0.57	0.80	11.70	0.000			
	Large	11	0.545	0.45	0.64	11.19	0.000			
	Tot. Betw. Overall	35	0.593	0.52	0.66	16.47	0.000			

**Table 4:** Tests for publication bias.

OLS Regression (Egger) Intercept *p*	0.095
Begg’s Test tau, *p*	−0.2303, 0.0517
Original g_mean	0.6799
Trim-and-Fill g_mean	0.7269
95% CI (Trim-and-Fill)	[0.6696, 0.7841]
Estimated Missing Studies	1

**Table 5:** Rosenthal’s fail-safe number calculations.

Z-value for observed studies	16.3602
*p*-value for observed studies	0.0000
Alpha	0.05
Tails	two-tailed
Z for alpha	1.96
Number of observed studies	35
Fail-safe N	2404

## Data Availability

The data is publicly available at https://doi.org/10.17605/OSF.IO/BQZ7W (accessed on 10 June 2026).

## Supplementary Materials

The following supporting information can be downloaded at https://www.mdpi.com/article/10.3390/jintelligence14060109/s1. Table S1: PRISMA 2020 checklist.

## Appendix A. The Meta-Analysis Data Collection Form

**Author(s) and Year**	**Grade/Class**	**Subjects**	**Durations of Implementation**	**Sample Size**
Alneyadi and Wardat (2024)	High School	Science	>3 months	Large Sample
Lin and Ye (2023)	Secondary	Science	>1, ≤4 weeks	Small Sample
Hwang et al. (2020a)	Primary	Maths	≤5 h	Large Sample
Henkel et al. (2024)	Primary + Secondary	Maths	>3 months	Large Sample
Erümit (2014)	High School	Maths	>3 months	Middle Sample
Cui et al. (2018)	Secondary	Maths	>1, ≤7 days	Large Sample
Hakiki et al. (2023)	University	Technology/Engineering	>3 months	Middle Sample
Li (2023)	University	Technology/Engineering	>1, ≤2 months	Middle Sample
Sun et al. (2024)	University	Technology/Engineering	>1, ≤2 months	Middle Sample
Fan et al. (2025)	University	Technology/Engineering	>3 months	Large Sample
Pathania et al. (2025)	Primary	Maths	>1, ≤2 months	Middle Sample
Ait Baha et al. (2024)	Secondary	Technology/Engineering	>1, ≤7 days	Middle Sample
El Fathi et al. (2025)	University	Technology/Engineering	>1, ≤2 months	Large Sample
S. Y. Chen et al. (2025)	University	Technology/Engineering	>1, ≤2 months	Middle Sample
Karaman and Goksu (2024)	Primary	Maths	>1, ≤2 months	Small Sample
Forero and Herrera-Suárez (2023)	University	Science	>3 months	Middle Sample
Keles (2007)	University	Maths	>1, ≤2 months	Middle Sample
Aydın (2023)	High School	Maths	>1, ≤2 months	Middle Sample
Bahçeci and Gürol (2016)	University	Technology/Engineering	>1, ≤2 months	Middle Sample
Lu et al. (2021)	University	Technology/Engineering	>1, ≤4 weeks	Middle Sample
Eryılmaz and Adabashi (2020)	University	Technology/Engineering	>1, ≤2 months	Small Sample
Yin et al. (2020)	University	Technology/Engineering	>1, ≤4 weeks	Middle Sample
Kömürcü (2025)	Secondary	Science	>1, ≤4 weeks	Small Sample
C. H. Chen and Chang (2024)	Secondary	Science	>1, ≤4 weeks	Large Sample
Johnson et al. (2024)	University	Technology/Engineering	>1, ≤7 days	Small Sample
Alneyadi and Wardat (2023)	High School	Science	>2, ≤3 months	Large Sample
Beltozar-Clemente and Díaz-Vega (2024)	University	Science	>3 months	Large Sample
Kosar et al. (2024)	University	Technology/Engineering	>3 months	Large Sample
Lee et al. (2024)	University	Science	>2, ≤3 months	Middle Sample
Wahba et al. (2024)	University	Maths	>1, ≤4 weeks	Middle Sample
Wu et al. (2024)	High School	Maths	>1, ≤4 weeks	Middle Sample
Çakir (2019)	University	Technology/Engineering	>3 months	Middle Sample
Yang et al. (2023)	University	Science	≤5 h	Small Sample
A. Y. Huang et al. (2023)	University	Technology/Engineering	>2, ≤3 months	Large Sample
Bai et al. (2025)	University	Technology/Engineering	>3 months	Middle Sample
